# BODY COMPOSITION AND FALL RISK IN MUTUALLY EXCLUSIVE CATEGORIES OF PHYSICAL ACTIVITY IN OLDER WOMEN

**DOI:** 10.1093/geroni/igad104.1952

**Published:** 2023-12-21

**Authors:** Renoa Choudhury, Joon-Hyuk Park, Chitra Banarjee, Ladda Thiamwong, Rui Xie, Jeffrey Stout

**Affiliations:** University of Central Florida, Orlando, Florida, United States; University of Central Florida, Orlando, Florida, United States; University of Central Florida, Orlando, Florida, United States; University of Central Florida, Orlando, Florida, United States; University of Central Florida, Orlando, Florida, United States; University of Central Florida, Orlando, Florida, United States

## Abstract

Both physical activity (PA) and sedentary behavior (SB) have been independently linked with health outcomes in older adults. However, limited research has been done to investigate how different combinations of these behaviors influence their body composition and functional balance. This cross-sectional study examined the associations of mutually exclusive categories of PA and SB with body composition and fall risk in older women. Accelerometer-measured PA (ActiGraph GT9X), body composition (Bioelectrical Impedance Analysis) and fall risk data (BTrackS Balance & sit-to-stand) were collected from 91 community-dwelling older women. Participants were grouped into four behavioral categories: active-low sedentary, active-high sedentary, inactive-low sedentary and inactive-high sedentary. Active was defined as ≥150 minutes of moderate-to-vigorous PA (MVPA) per week (inactive: < 150 minutes/week of MVPA) and low sedentary was denoted as residing in the first tertile of sedentary-to-light PA time ratio (high sedentary: residing in the remaining tertiles). Results from linear regression analysis showed that, compared to inactive-high sedentary group, lower body fat mass index (BFMI), higher skeletal muscle mass index (SMMI), lower appendicular fat mass index (AFMI) and higher sit-to-stand (STS) scores were found in both active-low sedentary (BFMI: p=.002; SMMI: p=.017; AFMI: p=.003; STS: p=.014) and inactive-low sedentary participants (BFMI: p=.007; SMMI: p=.014; AFMI: p=.005; STS: p=.031), while active-high sedentary participants only showed lower AFMI (p=.037). Our findings suggest PA interventions integrating approaches for achieving sufficient MVPA and reducing SB concurrently might promote healthy body composition and reduce fall risk among older adults.

